# The role of Ca^2+^ signalling in the pathology of exocrine pancreas

**DOI:** 10.1016/j.ceca.2023.102740

**Published:** 2023-06

**Authors:** Julia V. Gerasimenko, Oleg V. Gerasimenko

**Affiliations:** Cardiff School of Biosciences, Cardiff University, Museum Avenue, Cardiff, Wales, CF10 3AX, United Kingdom

**Keywords:** Calcium signaling, Cell death, Acute pancreatitis, Orai1/CRAC, Galactose, Bcl-2 BH4 domain, Pancreatic stellate cells, Pancreatic macrophages

## Abstract

Exocrine pancreas has been the field of many successful studies in pancreatic physiology and pathology. However, related disease - acute pancreatitis (AP) is still takes it toll with more than 100,000 related deaths worldwide per year. In spite of significant scientific progress and several human trials currently running for AP, there is still no specific treatment in the clinic. Studies of the mechanism of initiation of AP have identified two crucial conditions: sustained elevations of cytoplasmic calcium concentration (Ca^2+^ plateau) and significantly reduced intracellular energy (ATP depletion). These hallmarks are interdependent, i.e., Ca^2+^ plateau increase energy demand for its clearance while energy production is greatly affected by the pathology. Result of long standing Ca^2+^ plateau is destabilisation of the secretory granules and premature activation of the digestive enzymes leading to necrotic cell death. Main attempts so far to break the vicious circle of cell death have been concentrated on reduction of Ca^2+^ overload or reduction of ATP depletion. This review will summarise these approaches, including recent developments of potential therapies for AP.

## Highlights

• Ca^2+^ overload and ATP depletion are the main pathological processes in AP.
• The CRAC channel is the most attractive therapeutic target to reduce excessive Ca^2+^ entry.
• Modulation of IP_3_Rs and RyRs can efficiently inhibit pathological Ca^2+^ release.
• Energy supplementation can effectively reduce ATP depletion in AP.

## Graphical abstract

**Figure:**
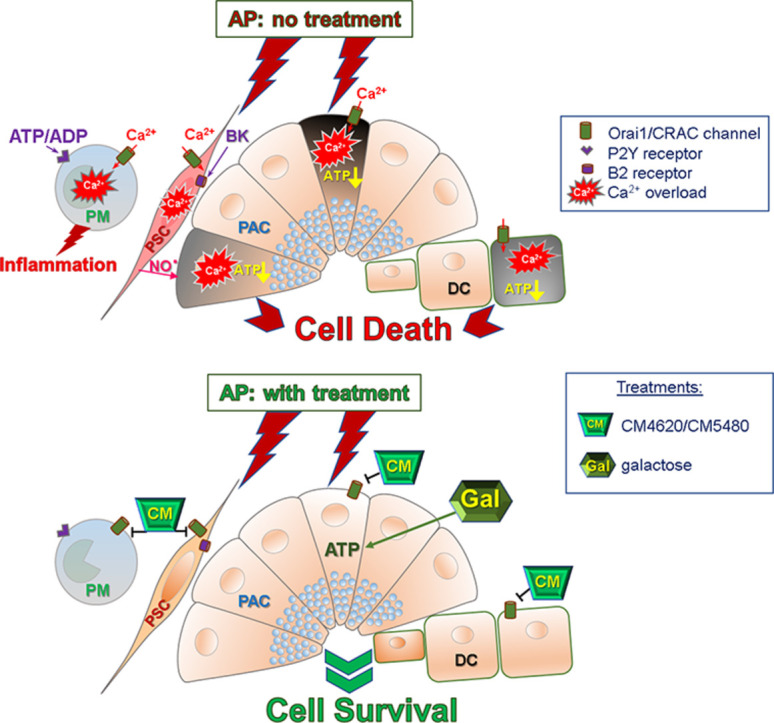

## 1 Introduction

Ca^2+^ signalling plays an important role in orchestrating the primary purpose of the exocrine pancreas to secrete essential digestive enzymes and fluids [1], [2], [3]. The central role in the key function of this gland belongs to pancreatic acinar cells (PACs). They populate up to 80% of the main tissue and are responsible for the initiation of pancreatic disorders such as acute pancreatis (AP). The disease can progress to chronic pancreatis (CP) that in turn significantly increases the risk of pancreatic cancer. Pancreatic ductal adenocarcinoma is characterised by a poor survival prognosis and a high mortality, with the projection to become the second leading cause of cancer-related deaths worldwide by 2030 [4]. The risk of cancer development following AP is 2.5-fold [5]; this makes this disease particularly dangerous. AP is a highly inflammatory and deadly disease on its own (>100,000 deaths in 2019 worldwide) [6] with mortality rates up to 30% in severe cases [6], [7], [8]. The two main causes of AP are alcohol abuse due to the toxic action of alcohol metabolites and biliary disease where bile duct blockage due to gallstones could lead to concentrated bile reflux into the pancreatic duct, affecting the cells of the organ. Both alcohol metabolites and bile acids act as AP inducing agents triggering the aberrant Ca^2+^ signalling in cells of the exocrine pancreas, primarily in PACs [9]. However, other cells within the same organ such as pancreatic stellate cells (PSCs), pancreatic duct cells, neurons and cells of innate and adaptive immunity also contribute to AP development through amplification of the inflammatory process [10]. For example, under physiological conditions, duct cells supplement pancreatic juice with bicarbonate and fluids and direct its flow towards the duodenum. However, their function is affected by alcohol metabolites and high concentrations of bile in AP, making this cell type particularly important in orchestrating disease development. The aim of this review is to discuss the role of Ca^2+^ signalling in the physiological and pathological processes in the cells of exocrine pancreas with the purpose of searching of potential therapeutic targets.

## 2 The role of Ca^2+^ signalling in the physiological function of pancreatic acinar cells

Under physiological conditions the exocrine pancreas supplies digestive (pro)enzymes and fluids to match daily dietary food consumption [3]. PACs, the most abundant cell type in the gland, are highly polarised with clearly defined basal and apical parts where the latter is packed with zymogen-containing secretory granules. PACs are assembled into basket-like acinar units and secrete acidic granular content by exocytosis at the apical membrane into a common lumen that is linked with the pancreatic duct. Duct cells supplement pancreatic juice with bicarbonate to neutralise the acidic pH of acinar secretion and navigate it further to the duodenum, where pancreatic enzymes become fully activated and capable to digest food [3].

### 2.1 Ca^2+^ release from intracellular stores

**Fig. 1:**
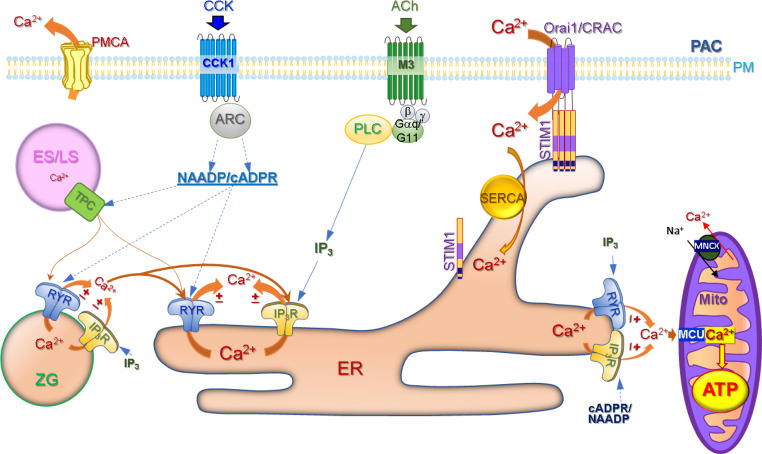
Schematic diagram presents the main physiological aspects of Ca^2+^ signalling in pancreatic acinar cells (PACs). Secretagogues acetylcholine (ACh) and cholecystokinin (CCK) act on G-protein coupled receptors, muscarinic M3 and CCK1, respectively. Stimulation of M3 receptor by ACh is linked to Phospholipase C (PLC) -dependant generation of inositol trisphosphate (IP_3_) and Ca^2+^ release from the ER through the IP_3_ receptor (IP_3_R) channel. The CCK1 receptor stimulated by CCK is coupled to ADP ribosyl cyclase (ARC) activation leading to production of cyclic ADP-ribose (cADPR) and nicotinic acid adenine dinucleotide phosphate (NAADP). They both are involved in Ca^2+^ release from the ER through the ryanodine receptor (RyR). IP_3_Rs and RyR are sensitised by Ca^2+^. NAADP also activates two pore channels (TPC) mainly located on the endosomal/lysosomal (ES/LS) membranes. Ca^2+^ released from TPC can be amplified by IP_3_Rs and RyR located in zymogen granules (ZG) and the ER. Ca^2+^ release from the ER is sensed by the mitochondrial Ca^2+^ uniporter (MCU), which is responsible for Ca^2+^ influx into the mitochondrial matrix that is linked to Ca^2+^-dependant generation of ATP by a stimulus-metabolism process. Any excess of Ca^2+^ in the mitochondrial matrix is removed by the Na^+^/Ca^2+^ exchanger (MNCX). Ca^2+^ release from the ER and subsequent reduction of Ca^2+^ within the ER lumen triggers the translocation and binding of the Stromal Interaction Molecule 1 (STIM1) with Orai1/Ca^2+^ release activated Ca^2+^ (CRAC) channel on the plasma membrane of PACs leading to Ca^2+^ influx through this channel and replenishment of the ER Ca^2+^ store with the help of Sarco Endoplasmic Reticulum Ca^2+^ ATPase (SERCA). Excessive cytosolic Ca^2+^ is pumped out of the cell by the plasma membrane Ca^2+^ ATPase (PMCA). Adapted from Petersen et al. 2021 [3].

Pancreatic acinar cell secretion is mediated by Ca^2+^ signalling pathways where the main secretagogues, the neurotransmitter acetylcholine (ACh) and hormone cholecystokinin (CCK) [3], activate their specific receptors, namely, muscarinic type 3 (M3) and CCK1, respectively, in a receptor-stimulus coupling manner (Fig. 1). ACh mediated Ca^2+^ release from the internal stores through inositol trisphosphate receptors (IP_3_Rs) type 2 and 3 evoke short-lasting local Ca^2+^ spikes in the apical region of the cell, triggering the apical secretion of zymogens by exocytosis. Stimulation of the CCK1 receptor by CCK is coupled to the activation of ADP ribosyl cyclase (ARC) leading to the production of cyclic ADP-ribose (cADPR) and nicotinic acid adenine dinucleotide phosphate (NAADP) [11]. Both Ca^2+^ releasing messengers are involved in the modulation of the ryanodine receptor (RyR), resulting in cytosolic Ca^2+^ oscillations and secretion of zymogens. Ca^2+^ oscillations induced by ACh and CCK also open Ca^2+^-dependant Cl- channels (TMEM16A/ANO1) in the apical region of the cell promoting fluid secretion into the acinar lumen [12,13].  Both IP_3_Rs and RyRs are Ca^2+^ sensitive, therefore their open state probability is increased with each Ca^2+^ spike initiating Ca^2+^-induced Ca^2+^ release [14]. However, with further local increase of Ca^2+^ to a specific level, calcium ions ultimately have an inhibitory effect on IP_3_Rs and RyRs, bringing the system to a resting state [15], [16], [17].

### 2.2 ATP production: glycolysis and mitochondrial Ca^2+^ handling

The secretion of digestive enzymes is a highly energy demanding process requiring the generation of a sufficient amount of ATP by mitochondria [3]. Glucose plays a central role as a cellular energy supply. It enters glycolysis by conversion to glucose-6-phosphate with the help of hexokinases. This is the first step in the glycolytic chain leading to production of pyruvate that fuels mitochondrial ATP production, which is a Ca^2+^-dependant process. During physiological stimulation of PACs with ACh or CCK, peri‑granular mitochondria help to maintain local Ca^2+^ oscillations in the apical region by transferring Ca^2+^ from the cytosol across the inner mitochondrial membrane (IMM) to the mitochondrial matrix through the pore-forming Ca^2+^ uniporter (MCU). In the matrix, Ca^2+^ stimulates the Krebs cycle enzymes involved in the stimulus-metabolism process of ATP production [3,18] (Fig. 1). The Ca^2+^ excess is removed from the matrix by the Na^+^/Ca^2+^ exchanger.

### 2.3 Store operated Ca^2+^ entry

Reduction of the Ca^2+^ level within the ER lumen during Ca^2+^ signalling is sensed by the EF-hands of Stromal Interaction Molecule 1 (STIM 1) leading to the activation, oligomerisation and translocation of STIM1 to the plasma membrane junctions (puncta). At the plasma membrane junctions STIM1 binds and opens Store Operated Ca^2+^ Entry (SOCE) channels, such as Orai1/Ca^2+^ release activated Ca^2+^ (CRAC) channels (Fig. 1) [19]. Opening of these channels allows Ca^2+^ to flow into the cell to replenish the ER Ca^2+^ content with the help of Sarco/Endoplasmic Reticulum Ca^2+^ ATPase (SERCA). Any cytosolic Ca^2+^ excess is removed by the plasma membrane Ca^2+^ ATPase (PMCA) (Fig. 1) [20,21].

## 3 Involvement of different cell types of the exocrine pancreas in generating acute pancreatitis

### 3.1 Pancreatic acinar cells

**Fig. 2:**
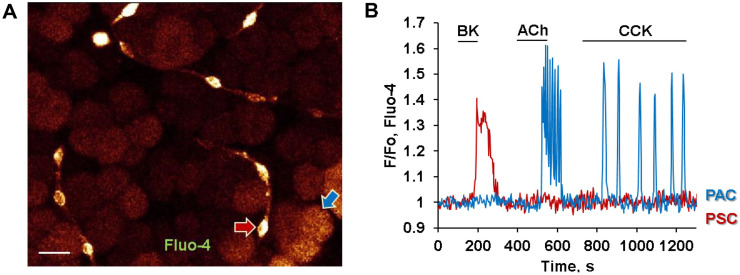
**A.** Pancreatic stellate cells (PSC) are located in the periacinar space within the pancreatic tissue. Scale bar represents 10 µm. **B.** Representative traces from the same experiment where pancreatic lobules were stimulated with bradykinin (BK) (0.5 nM), ACh (20 nM), and CCK (5 pM). PSC (red arrow in A and red trace in B) produced a typical Ca^2+^ response to BK application but not to other agonists that are specific for PACs (blue arrow in A and blue trace in B). Pancreatic lobules were loaded with Fluo-4 AM.

PACs are the most extensively studied cells of the exocrine pancreas due to their very high presence within pancreatic tissue [1], [2], [3]. However, their morphology and properties are somewhat different when they are part of the tissue. The isolation of PACs is quite a damaging process that results in substantial cell death (up to 10%) and release of various pancreatic proteases, radicals and other factors from affected neighbouring cells. While fresh isolation of PACs is a very valuable technique to study pancreatic physiology and pathology, results obtained using such models should be considered cautiously against the potential activation of a number of extracellular receptors during isolation. Nevertheless, the most valuable results defining our knowledge about exocrine pancreatic physiology and pathology have been obtained using an isolated preparation [9,[22], [23], [24], [25], [26], [27]]. The short life time of PACs after isolation is another limiting factor that complicates long-term studies such as protein expression or sRNA. Historically, short-term pancreatic cultures have been used for such experiments [28]. Unfortunately, none of the existing cultured cell lines are accurate models of pancreatic physiology. Recently, early experiments with pancreatic tissue [29] have been reincarnated using modern confocal and two-photon microscopy [30,31].  With regards to PACs, their typical Ca^2+^ responses in freshly isolated cell preparations were essentially confirmed in pancreatic lobules *in situ* [10,31]. The unique polarisation of PACs and the typical physiological and pathological effects displayed essentially the same patterns [10,31]. The individual cell morphology, including apical localisation of secretory granules surrounded by the mitochondrial protective belt, was confirmed in the tissue, adding only a striking 3D perspective. Stimulation of PACs in the lobule with the “classical” agonists ACh and CCK has resulted in Ca^2+^ signals (Fig. 2, blue trace) very similar to those demonstrated previously in isolated cells [1], [2], [3].

Physiological Ca^2+^ signalling in PACs promotes their key functions; however, the excessive and sustained Ca^2+^ elevations caused by pathological agents initiate AP [3]. Mechanisms of pathological Ca^2+^ signalling in PACs will be discussed in the Section 4 of this review.

### 3.2 Pancreatic duct epithelial cells

The transport of pancreatic enzymes and fluids secreted from PACs is accomplished by the ductal network, responsible for delivery of pancreatic juice to the duodenum [3]. The fundamental role of pancreatic ductal epithelial cells (PDECs) is to neutralise the acidic milieu of acinar secretion by releasing bicarbonate (HCO_3_^−^).  The main source of HCO_3_^−^ in PDECs comes from the interstitial fluid via the Na^+^/HCO_3_^−^ co-transporter located at the basolateral membrane. More HCO_3_^−^ is generated by carbonic anhydrases from CO_2_ and H_2_O in the cytosol. The bicarbonate release from PDECs to the lumen is mediated by anion transporters such as SLC26A3 and SLC26A6 and the cystic fibrosis transmembrane conductance regulator (CFTR) located on the basolateral membrane [32]. The hormone secretin regulates the CFTR by stimulating adenylate cyclase production of cAMP. Ca^2+^ signalling has been shown also to be involved in bicarbonate secretion from PDECs mediated by ACh and ATP [33]. In AP the aberrant Ca^2+^ signalling in PDECs results in inhibition of HCO3- secretion and mitochondrial damage with loss of ATP production leading to cell death [34,35].

### 3.3 Pancreatic stellate cells

More than two decades ago some very important discoveries were made in relation to a cell type present in relatively small amounts (<5%) in pancreatic tissues, namely PSCs [36,37]. Remarkable differences and new effects have been recently found in pancreatic tissue segments *in situ* [29] for this cell type.

**Fig. 3:**
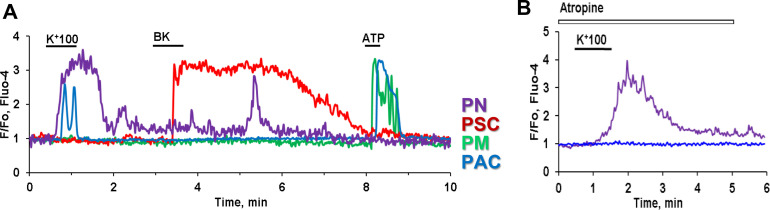
Depolarisation of neurons by high *K*^+^ (100 nM) results in Ca^2+^ signals in PACs but not in stellate (PSCs) or immune cells in the same lobule. A. Representative traces of Ca^2+^ responses in pancreatic neuron (purple trace), PAC (blue trace), PSC (red trace) and immune cell (green trace) after subsequent stimulation of the pancreatic lobule with 100 nM KCl, 1 nM bradykinin (BK) and 100 µM ATP. PAC produce Ca^2+^ signal to high *K*^+^ application with a delay as compared to the Ca^2+^ response from a neuron. B. Ca^2+^ signal to high *K*^+^ is blocked by 10 µM atropine in PAC but not in the neuron. Pancreatic lobules were loaded with Fluo-4 in AM form. Modified from Gryshchenko et al. 2018 [10].

PSCs are located at the periphery of acinar baskets (Fig. 2A) [31]. They are presented as a network of small, elongated cells with protrusions that often branch and connect with other neighbouring PSCs at different focal planes of the pancreatic tissue [31] (Fig. 2A). The physiological role of PSCs is not well understood. They have been characterised as quiescent cells that perform a housekeeping role by providing the structural integrity of the extracellular matrix and supplying the tissue with lipids [38]. These cells can be identified in the pancreatic tissue *in situ* using visualisation of vitamin A containing lipid droplets in their cytoplasm [31]. Until relatively recently, PSCs have been studied predominantly in cell cultures [39], where they change from a quiescent to an activated state with substantial changes of their properties including their sensitivity to stimuli [30]. For example, cultured PSCs displayed more than three orders of magnitude less sensitivity to bradykinin (BK), as compared to PSCs in pancreatic lobules *in situ* [10,31,40]. BK is a vasoactive nonapeptide that originates from kininogens by tissue and plasma kallikrein; the latter is produced by exocrine glands including the pancreas [41]. Physiological concentrations of BK are tightly controlled at a very low level (∼40–80 pM) [42]. BK is involved in blood pressure regulation and in inflammatory reactions due to its ability to increase vascular permeability and vasodilation [43], [44], [45]. PSCs are sensitive to as low as ∼ 50 pM BK with a maximum sensitivity in the presence of 1nM BK [10,31]. At such a low concentration, BK is acting on the B2 receptor in PSCs producing a biphasic Ca^2+^ response (Figs. 2B and 3A, red traces) that result in the cellular signalling pathway of nitric oxide (NO) production [10,31,46]. The initial phase and the BK response itself are sensitive to inhibitors of IP_3_R signalling such as caffeine, 2-aminoethoxydiphenyl borate (2-APB) and also the PLC inhibitor U73122. The second phase of the BK response is dependant on extracellular Ca^2+^ influx through SOCE channels that include Orai1/CRAC channels. The treatment of lobules with blockers of Orai1/CRAC channels with GSK 7975A (GlaxoSmithKline) or CM4620 (CalciMedica) completely abolished the second plateau phase without affecting the IP_3_R-dependant first (spike) phase in PCSs [47], [48]. Other cell types in the exocrine pancreatic tissue do not produce any responses at such low concentrations of BK (Fig. 3).  In the presence of increased BK levels during some pathological conditions including AP, PSCs are shown to be activated, and at the same time become less sensitive to picomolar doses of BK. They start to produce Ca^2+^ signals only at 1nM with a maximum sensitivity at 30nM BK, most likely, a result of B2 receptor desensitisation in AP [10]. Furthermore, PSCs in pancreatic tissue taken from mice with an experimental *in vivo* model of alcohol-induced AP, display sensitivity to the BK B1 receptor agonist Sar-Des-BK. Interestingly, PSCs in tissue from control animals do not exhibit any responses to B1 agonists, highlighting the role of PSCs in AP development [10]. This is in contrast to the belief that activated PSCs undergo changes of phenotype to myofibroblast-like with increased proliferation and remodelling and support tissue transformation to chronic pancreatitis and pancreatic cancer [37,49].

### 3.4 Pancreatic neurons

The pancreatic lobule preparation allowed us also to study other less represented cells of the exocrine pancreas, namely the neurons and immune cells [10,47] (Fig. 3, purple and green traces, respectively). Ca^2+^ signals have been observed in neurons located in pancreatic lobules as a result of membrane depolarisation by the application of high *K*^+^ concentrations (100mM) (Fig. 3). With some delay, non-excitable PACs in the neighbourhood have also produced Ca^2+^ responses in response to a neurotransmitter such as ACh, released from neurons by depolarisation (Fig. 3). These responses in PACs can be blocked by atropine [10] (Fig. 3B). While this is not a surprising result, this area requires some thorough further investigation, particularly with regard to the pathological aspects of intercellular communications. Pancreatic neurons are responsible for severe pain in AP patients representing an important prime hallmark of the disease [50].

### 3.5 Pancreatic macrophages

**Fig. 4:**
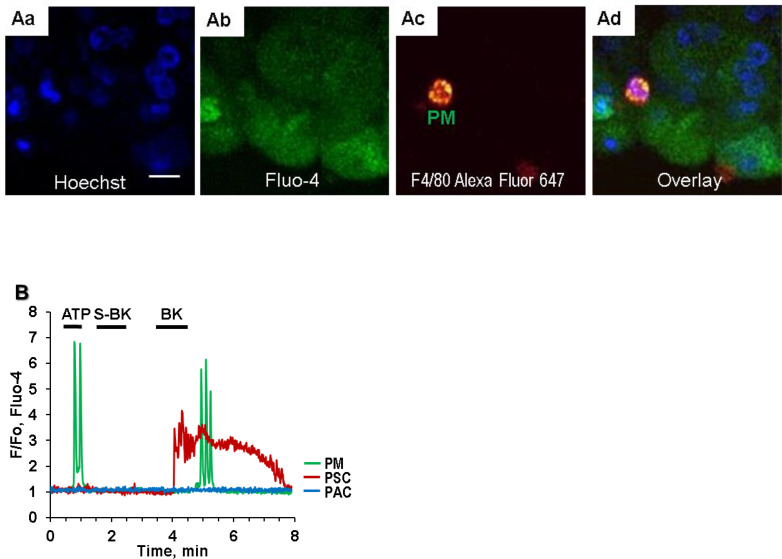
Ca^2+^ signalling in pancreatic macrophages. A. Identification of pancreatic macrophage in lobules using staining with fluorescent nuclear indicator Hoechst 33342 (Aa), Fluo-4 (Ab), fluorescently labelled antibody F4/80 conjugated with AlexaFluor 647 (Ac). Overlay of Aa, Ab and Ac is shown in Ad; scale bar 10 μm). B. A representative trace that demonstrates Ca^2+^ signals in pancreatic macrophage (green trace) and PSC (red trace) induced by 20 nM BK (with a clear delay in the macrophage). The macrophage was the only cell that produced a Ca^2+^ response to 10 µM ATP. Neither of the cells responded to application of 1 µM Sar-Des-BK. PAC (blue trace) did not produce any responses to the above agonists. Lobules were loaded with Fluo-4 AM. Modified from Gerasimenko et all. 2022 [48] and Gryshchenko et al. 2020 [47].

The immune cells of the exocrine pancreas have been a target for research due to their role in AP, chronic pancreatitis and pancreatic cancer [47,[51], [52], [53]]. The new method showed the presence of a relatively small number of ATP-sensitive cells in close proximity to some PSCs (Figs. 3A and 4A; green traces). Immunostaining with different surface antibodies of immune cells has shown that most of them are, in fact, pancreatic macrophages [47]. This cell type produces Ca^2+^ signals in response to ATP/ADP acting on purinergic receptors P2Y1 and P2Y13 that are linked to Ca^2+^ release from internal stores through IP_3_-dependant signalling [47,54]. In general, pancreatic macrophages *in situ* show the ability to respond to BK with much lower sensitivity than neighbouring PSCs. Ca^2+^ signals in macrophages are detectable from 3 to 30nM BK but with a substantial delay as compared to responses to lower concentrations 50 pM – 1nM in PSCs (Fig. 4B) [47]. Due to PAC necrosis in AP, elevated levels of ATP/ADP spilled out from necrotic acinar cells activate immune cells that in their turn mediate an inflammatory response within the tissue [3]. At the same time leakage of pancreatic acinar kallikrein is responsible for further damage to the tissue by producing high concentrations of BK that can act on PSCs and macrophages, exacerbating disease progression. In line with that suggestion [3], we have demonstrated recently that the density of immune cells significantly increased after induction of the experimental *in vivo* model of FAEE-AP in mice [47].

## 4 Abnormal Ca^2+^ signalling and hallmarks of acute pancreatitis

The highly tuned Ca^2+^ signalling regulation of physiological functions in the exocrine pancreas is, in fact, highly vulnerable due to the action of various pathological substances, for example, alcohol metabolites, bile acids and certain drugs. These agents, that apparently have nothing in common, exhibit similar abilities to disrupt the physiological function of the exocrine pancreas by interfering with Ca^2+^ release and Ca^2+^ entry mechanisms resulting in cytosolic and mitochondrial Ca^2+^ overload, inhibition of glycolysis, loss of cellular ATP and premature intracellular activation of digestive proteases such as trypsin [9,27,[55], [56], [57], [58], [59], [60]]. These events trigger pancreatic cells necrosis and tissue inflammation that are the main hallmarks of AP.

### 4.1 Alcohol induced acute pancreatitis

Alcohol binge drinking is often a cause of AP, also increasing the risk of transition of the disease to its chronic form [3]. In the pancreas, non-oxidative alcohol metabolites in combination with long-chain fatty acids are converted by carboxyl ester lipase (CEL) into fatty acid ethyl esters (FAEE) [61]. Accumulation of FAEE during alcohol intoxication has been found in many human vital organs such as liver, heart and brain but they are found in their highest amounts in the pancreatic parenchyma causing AP. Inhibition of CEL, which restricts formation of FAEE, has been shown to protect against toxic alcohol *in vitro* effects as well as in experimental *in vivo* mouse models of alcohol related AP [62]. Recently, FAEE have been proposed to serve as biomarkers for the diagnosis of alcohol-related AP [63]. It has been shown previously that one representative of FAEE, the palmitoleic acid ethyl ester (POAEE), can trigger AP (FAEE-AP) by activating Ca^2+^ release from intracellular stores through IP_3_R type 2 and 3 in PACs [57,62]. In the presence of POAEE, significant Ca^2+^ loss from the ER triggers excessive Ca^2+^ entry through SOCE channels such as Orai1/CRAC channels [27]. Since Ca^2+^ uptake into the ER is inhibited, due to ATP depletion, the uncontrolled Ca^2+^ overflow through CRAC channels leads to cytosolic and mitochondrial Ca^2+^ overload, exacerbating ATP loss. Sustained Ca^2+^ overload leads to vacuole formation [55] and intracellular activation of trypsinogen in the apical region of PAC. Under these pathological conditions, mitochondria are also overloaded with Ca^2+^ and unable to produce a sustainable amount of ATP, thereby failing to support removal of cytosolic Ca^2+^ excess by PMCA and SERCA. In the absence of ATP, PACs, overwhelmed by Ca^2+^overload, die by necrosis. During the process of necrotic cell rupture, active digestive enzymes such as the powerful protease trypsin are released from PACs into the interstitial milieu. The leakage of another pancreatic enzyme, kallikrein, is responsible for the increased production of blood pressure lowering nonapeptide bradykinin [10]. In its turn, bradykinin stimulates bradykinin type 2 (B2) receptors on neighbouring PSCs that wake up this cell type from the quiescent state. Activated PSCs produce cytokines and nitric oxide, amplifying the damaging effect from necrosis. ATP and ADP, leaked from necrotic cells, directly act on immune cells orchestrating the inflammatory processes in pancreatic tissue and surrounding organs [3]. Detrimental effects of alcohol and FAEE have been demonstrated in PDECs by inhibiting essential physiological functions of this cell type such as secretin-induced bicarbonate and fluid release into the duct lumen [32]. Both alcohol and FAEE cause a sustained elevation of cytosolic Ca^2+^ and mitochondrial dysfunction resulting in reduced ATP production in PDECs. There is growing evidence that the CFTR expression level, the process of protein folding and stability within the plasma membrane, are greatly affected by alcohol and its metabolites [64].

### 4.2 Bile induced acute pancreatitis

In biliary disease, blockage of the common bile duct by gallstones is one of the most common causes of AP [65]. As a result, bile with a high concentration of bile acids refluxes into the pancreatic duct resulting in the damage of pancreatic cells.

Therefore, PDECs are the first cell type facing the toxic effects of bile. It has been shown that a high concentration (1mM) of bile acids, such as chenodeoxycholate, perfused into isolated ducts, elicited sustained intracellular Ca^2+^ elevations in PDECs leading to mitochondrial damage, loss of cellular ATP and disruption of bicarbonate secretion and fluid transport [34,66]. The extracellular acidification due to the insufficiency of HCO3- release by PDECs has been associated with an increase in intraductal activity of trypsin, which leaks from necrotic PACs during AP. Trypsin has been shown to inhibit apical CFTR and anion exchangers by stimulating protease-activated receptor 2 (PAR2) at the luminal plasma membrane of PDECs [67]. Another action of trypsin is the disruption of tight cell junctions, significantly amplifying the pathogenesis of AP.

Bile acids have several targets in PACs. It has been reported that two transport mechanisms, the Na-taurocholate co-transporting polypeptide, expressed mainly in the luminal membrane, and the organic anion transporting peptide 1, are involved in accumulation of bile acids in PACs [68,69]. However, bile acids have also been shown to act on the G protein coupled bile acid receptor 1 (Gpbar1) [69]. The intracellular Ca^2+^ signalling pathway activated by bile has been shown to depend on IP_3_Rs and RyRs opening because blockers of these channels inhibit bile acids’ effect on intracellular Ca^2+^ release.

However, bile duct obstruction with gallstones also increases the intraductal pressure that can mechanically activate the nonselective cation Piezo1 channels expressed on the plasma membrane in many cell types including PACs [70]. Piezo1 are permeable to Na^+^, *K*^+^, Ca^2+^ and Mg^2+^ when stimulated by mechanical pressure, for example, during certain types of abdominal surgery or by the pharmacological activator Yoda1 [71], [72], [73]. Activation of Piezo1 by Yoda1 or fluid sheer stress representing intraductal pressure elicits sustained cytosolic Ca^2+^ rise, mitochondria depolarisation, improper trypsinogen activation and necrosis in PACs, manifesting the hallmarks of AP. Interestingly, Piezo1 does not work alone but in a relationship with another non-selective cation transient receptor potential vanilloid-type 4 (TRPV4) channel that also mediates influx of external Ca^2+^. Expression of TRPV4 has been found in many cell types and tissues including PACs. A small initial transient Ca^2+^ influx through Piezo1 activates phospholipase A2 which catalyses the production of the endogenous agonist of TRPV4, 5,6-Epoxyeicosatrienoic acid (5,6-EET), which in turn amplifies Ca^2+^ entry through TRPV4 [73]. These results imply both channels are incriminated in the development of pressure related AP pathology.

### 4.3 Asparaginase-induced acute pancreatitis

AP is also known to be a side effect of treatment with the drug l-asparaginase in up to 10% of patients with Childhood Acute lymphoblastic Leukaemia (ALL) [74]. l-asparaginase has been used for many years as a very effective treatment for ALL due to the effective ability of the drug to starve cancer cells of asparagine [75]. Asparagine synthesis in cancer cells is negligible; instead, they take up this important nutrient from the blood supply. The ability of l-asparaginase to break down asparagine in blood, makes it unavailable for cancer cells which leads to their starvation and death. However, in severe cases of l-asparaginase-induced AP (AAP) the drug treatment has to be discontinued leaving patients with very limited options to treat the leukaemia. Therefore, to prevent the side effect of l-asparaginase, there is a pressing need to investigate the mechanism in detail and identify new therapeutic targets [59,60].

The effect of l-asparaginase in PACs mainly relies on the stimulation of PAR2 leading to Ca^2+^ release from internal stores followed by excessive Ca^2+^ entry through Orai1/CRAC channels. Therefore, it carries distinctive similarities to the actions of alcohol metabolites and bile, also resulting in intracellular Ca^2+^ overload in PACs and affecting ATP production. Without a sustainable ATP supply, cells die by necrosis, leading to inflammatory processes in the pancreas and surrounding tissues [59,60]. Another similarity between the action of l-asparaginase, FAEE and bile is the inhibition of the hexokinases that makes glucose-6-phosphate unavailable in the first stage of the glycolysis pathway. This blocks glycolytic production of pyruvate, one of the main fuels for mitochondrial ATP synthesis [60]. We suggested an alternative - the natural carbohydrate galactose that does not require hexokinases to join and support glycolysis, therefore, it can act as an ideal oral energy supplement to protect PACs and the pancreas against AP [60,76].

### 4.4 COVID-19 and acute pancreatitis

It has been well established that AP can be caused by various viral infections such as mumps, Coxsackie B virus and hepatitis A virus [77,78]. With the current global pandemic of coronavirus disease 2019 (COVID-19) caused by the severe acute respiratory syndrome coronavirus 2 (SARS-CoV-2) there is growing evidence that the virus triggers many inflammatory diseases including several reported cases of associated AP [79], [80], [81], [82].

It has been shown that SARS-CoV-2 can enter host cells via receptor-mediated endocytosis that is triggered by the binding of its Spike glycoprotein subunit 1 (S1) to Angiotensin-Converting Enzyme-2 (ACE2) receptors [83], [84]. ACE2 receptors have been found to be expressed throughout the gastrointestinal tract including the exocrine pancreas [85]; however, until recently little was known about the distribution of these receptors in pancreatic cell types.

Taking advantage of the mouse pancreatic lobule preparation, we have recently demonstrated that S1 elicits Ca^2+^ responses in PSCs and macrophages with a minimum level of 70 nM causing 25% of PSCs and 33% of macrophages to respond, whereas levels of 600 nM caused all tested PSCs and macrophages to respond [48]. Interestingly, we did not observe Ca^2+^ signals to S1 from PACs *in situ* experiments. In addition, we noticed a considerable delay in the response to S1 by macrophages as compared to PSCs; stellate cells developed Ca^2+^ spikes 3.8 times faster (*P*<0.006). We have found that this difference in the initiation of Ca^2+^ signals between these cell types is due to S1-induced release of pro-inflammatory cytokine interleukin 18 (IL-18) by PSCs that in its turn stimulates Ca^2+^ signals in macrophages. The IL-18 binding peptide eliminates S1 responses in macrophages but does not interfere with S1-elicited Ca^2+^ signals in PSCs. COVID-19 has been associated with the “cytokine storm” that orchestrates inflammatory processes in the lungs, pancreas and other organs [86]. From our experiments we conclude that in the pancreas, non-immune PSCs can play an initiation role in S1-induced inflammation by stimulatory signals to the cells of innate immunity.

Store-operated Ca^2+^ entry plays an important role in S1 Ca^2+^ signals in PSCs and macrophages because removal of external Ca^2+^ abolished all S1-induced responses in macrophages [48]. Blockage of Orai1/CRAC channels with CM4620 (Auxora; CalciMedica) (10 µM) eliminated all Ca^2+^ signals in macrophages and 75% of Ca^2+^ signals in PSCs. These results suggest that S1 activates Orai1/CRAC channels, known to be highly involved in AP, therefore, S1 alone could trigger pancreatic injury in infected individuals or potentiate effects of other AP-inducing agents. In conclusion, Orai1/CARC channel have become a justified therapeutic target for treating inflammation and reducing the risk of AP in patients with COVID-19 [48].

## 5 Potential therapeutic avenues

### 5.1 Regulation of Ca^2+^ release

As mentioned above, the main function of the exocrine pancreas is to produce and deliver pancreatic juice to the small intestine to assist in breaking down components of our food into absorbable essential nutrients. The physiological process of pancreatic juice secretion is controlled by Ca^2+^ signalling that includes secretagogues binding to their G-protein coupled receptors leading to production of intracellular Ca^2+^ messengers and opening of IP_3_Rs, RyRs and two pore channels that allow Ca^2+^ to be released into the cytosol from internal stores such as the ER and acidic Ca^2+^ stores [3].

However, in AP the induction of Ca^2+^ release is different from the classical stimulus-secretion coupling described above. Instead, AP-inducing agents mediate excessive Ca^2+^ release from internal stores, which becomes a crucial step in the subsequent induction of cellular Ca^2+^ overload, loss of ATP and premature activation of digestive proteases leading to necrotic cell death. We have previously shown that IP_3_Rs are responsible for the excessive intracellular Ca^2+^ release induced by agents causing AP (*i.e.*, alcohol, bile acids, asparaginase) and showed that genetic deletion of IP_3_Rs sub-types 2 and 3 prevented cytosolic Ca^2+^ overload and intracellular protease activation [25,57]. We have also demonstrated that the sustained bile - induced intracellular Ca^2+^ rise is dependant on RyR opening, which has been linked to activation of pro-inflammatory processes [25,87].

It appears that the regulation of Ca^2+^ release during pathological cell stimulation is one of several attractive targets in the research of novel therapeutic avenues for the development of AP treatment.

Previously, we discovered an intrinsic protective mechanism in the form of the intracellular Ca^2+^ sensor calmodulin (CaM) that regulates the opening of IP_3_Rs and RyRs by mediating significant conformational changes in both receptor channels, which impacts their function [58,88]. Both Ca^2+^ free CaM (ApoCaM) and Ca^2+^-occupied CaM show high affinity to RyRs and can inhibit both RyR1 and RyR2 at high Ca^2+^ concentrations, [89] which would be ideal for preventing Ca^2+^ overload during AP. Ca^2+^ dependant inhibition of IP_3_Rs by CaM has also been reported with identification of several CaM binding sites [88,90]. It has been found that the cell-permeable Ca^2+^-like peptide 3 (CALP3) can bind and activate CaM using a similar mechanism to Ca^2+^. Therefore, we have employed this approach to boost the effects of CaM in the regulation of IP_3_Rs and RyRs under pathological experimental conditions [58]. We have found that pre-treatment of the permeabilized and intact isolated PACs with CALP3 (100µM) abolished the toxic effects of high concentrations of alcohol [58,91]. With modification of CALP (mCALP) we have shown that 0.1µM mCALP was sufficient to reduce necrosis in PACs, induced by the alcohol metabolite palmitoleic acid ethyl ester (POAEE) (100µM), which makes this an attractive avenue for the development of AP treatment by regulation of intracellular Ca^2+^ releasing channels [92].

Another direction, currently quickly developing, is the Bcl-2 dependant regulation of Ca^2+^ release. The anti-apoptotic members of B-cell lymphoma (Bcl)−2-proteins, Bcl-2 and Bcl-extra-large (Bcl-XL) have been established as endogenous negative regulators of IP3Rs and RyRs [93,94].  We have demonstrated previously that Bcl-2 is involved in the regulation of calcium extrusion in PACs [95], an effect that would also be useful in the inhibition of Ca^2+^ overload. As for Ca^2+^ release, it was shown that the N-terminal Bcl-2 homology (BH) 4 domain directly interacts with the central, modulatory region and ligand-binding region of IP3R and inhibits the opening of the channel [93]. Similarly to Bcl-2, the Bcl-XL protein functions as an inhibitor of IP3R channels as shown both in intact living cells and in single-channel recordings [96]. Both Bcl-2 and Bcl-XL have been reported to inhibit Ca^2+^ release through RyR channels via binding to their central, modulatory domain [97,98]. To emulate the action of the BH4 domain of Bcl-2 and Bcl-XL, BH4 domain-derived peptides were employed to demonstrate the effectiveness of the BH4 domain in inhibition of IP_3_Rs in different cell types [93,98]. We have recently shown that both the BH4 domain derived peptides (50µM) of Bcl-2 and Bcl-XL significantly reduced excessive Ca^2+^ release from internal stores as well as PACs’ necrosis induced by the bile acid Taurolithocholic acid 3-sulfate (TLC-S), demonstrating a therapeutic potential of this approach [94].

### 5.2 Regulation of store operated Ca^2+^ entry

**Fig. 5:**
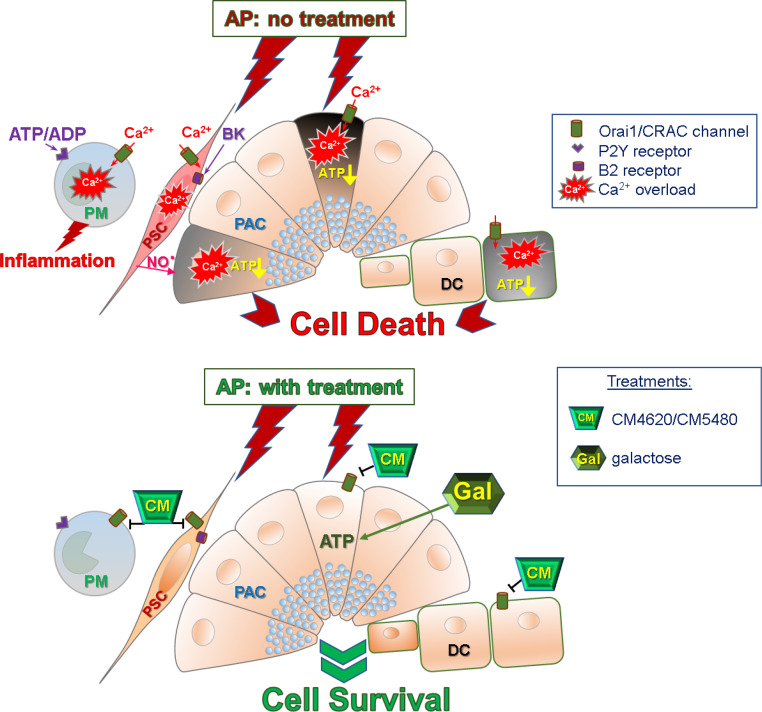
Schematic diagram representing mechanism of initiation of acute pancreatitis (AP) (top panel) and a summary of potential therapeutic interventions (lower panel). AP pathology involves Ca^2+^ overload due to excessive intracellular Ca^2+^ release followed by sustained Ca^2+^ entry through Orai1/CRAC channels in pancreatic acinar cells (PAC) causing loss of ATP, cell necrosis with the leakage of trypsin, kallikrein and ATP/ADP. Elevated levels of bradykinin (BK), produced by kallikrein, act on B2 receptors that are located in the plasma membrane of pancreatic stellate cells (PSCs) leading to Ca^2+^ signals generation, activation of nitric oxide synthase and release of nitric oxide (NO·). NO· and active trypsin potentiate necrosis in PACs, amplifying the primary effect of AP-inducing agents. ATP/ADP bind to P2Y1/P2Y13 purinergic receptors in pancreatic macrophages (PM), producing Ca^2+^ signals and activating production of proinflammatory cytokines. Abnormal Ca^2+^ signalling in duct cells (DC) inhibits bicarbonate secretion, exacerbating AP pathology. The lower panel depicts the effects of potential treatments in preventing pathological events in pancreatic tissue. Selective inhibition of Orai1/CRAC channels with the help of CalciMedica (CM) blockers (CM4620 for PACs, PSCs and PM; CM5480 for DC) controls the intracellular Ca^2+^ levels in all four cell types inhibiting the adverse effects of Ca^2+^ overload. Galactose provides an alternative energy source for ATP production, essential for recovery from Ca^2+^ overload and, therefore, supports cell survival.

It is becoming increasingly evident that Orai1/ CRAC channels are linked to a number of incurable human diseases including cancer, inflammatory bowel disease, rheumatoid arthritis and AP [99]. Despite the vital physiological role of Orai1/ CRAC channels in stimulus-secretion coupling in PACs, they become responsible for the fatal cytosolic Ca^2+^ overload in this cell type induced by AP-inducing agents such as alcohol metabolites, bile, and asparaginase [27,60,100]. The absence of external Ca^2+^ or pharmacological blockade of Ca^2+^ entry by Orai1 inhibitor GSK-7975A (GlaxoSmithKline) is found to be beneficial for PACs’ survival in preventing sustained Ca^2+^ elevation and protease activation induced by the non-oxidative alcohol metabolite POAEE [27] (Fig. 5). Our findings were confirmed by *in vivo* studies that have demonstrated the effectiveness of GSK-7975A and CM_128 (CM4620; CalciMedica) against pancreatic tissue injury using three experimental mouse models of AP, induced by intraperitoneal injections of alcohol and fatty acid mixture, retrograde pancreatic ductal injections of bile acids and hyperstimulation with the CCK analogue, cerulein [101]. Currently, the CalciMedica compound CM4620 (Auxora) is involved in clinical trials for AP, asparaginase-associated AP and for ventilated COVID‐19 acute respiratory distress syndrome [102], [103], [104]. However, Orai1/CRAC channels are also responsible for Ca^2+^ entry in other cells of the exocrine pancreas such as PSCs and resident immune cells [10,31,47]. In PSCs the second sustained Ca^2+^ entry phase of the BK-induced Ca^2+^ response is dependant on SOCE through Orai1 channels. The removal of external Ca^2+^ or presence of the Orai1 channel blocker GSK-7975A almost completely abolishes the sustained phase but does not prevent the initial IP_3_-dependant spike [10,31] (Fig. 5). Another cell type in the exocrine pancreas that also benefits from Orai1 inhibition are the pancreatic ductal cells. Recently, it has been found that a novel CalciMedica compound CM5480 is capable of reducing the Ca^2+^ overload in duct cells, induced by non-oxidative ethanol metabolites or bile acids *in vitro* and *in vivo,* by increasing cell survival and function under AP conditions [105,106] (Fig. 5).

Pancreatic immune cells represent major contributors of the inflammatory cascade during AP. The primary infiltration of immune cells to the injured organ is intended to resolve the damage produced by PACs' necrosis, with a view to promoting tissue recovery. However, in severe cases the pro-inflammatory processes will persist in acting as a lethal factor in AP [107]. SOCE has been implicated in the activation and proliferation of immune cells with production of reactive oxygen species (ROS) and cytokine release [31,108]. We have demonstrated recently that the treatment of pancreatic lobules with Orai1 channel blocker GSK 7975A (20µM) abolishes SOCE in pancreatic macrophages [47] (Fig. 5). Targeting Orai1/CRAC channels with CM4620 compound in freshly isolated neutrophils and human peripheral blood mononuclear cells (PBMC) resulted in reduction of ROS and cytokine production, respectively [109]. However, it has been shown that mice with a genetic deletion of Orai1 in PACs show a high rate of mortality - up to 70% within three weeks as a result of bacterial dysbiosis and reduction in antimicrobial secretion [110]. Moreover, individuals with Orai1 loss-of-function mutations are susceptible to a range of immunological and muscular diseases [111]. Further studies are necessary to determine the safety and consequences of the long-term administration of Orai1 inhibitors.

Pharmacological inhibition of Piezo1, TRPV4 and phospholipase A2 as well as knock out of both channels in mice have shown a protection against AP hallmarks suggesting these novel molecular targets could be highly important in future pressure-induced AP treatment. However, it is worth mentioning that embryonic knockouts of Piezo1 cannot survive due to defects in the vascular system [112], while loss-of-function mutations of Piezo1 induce generalised lymphatic dysplasia [113]. Therefore, TRPV4 could be an inappropriate target since it is highly expressed in many tissues and involved in control of key physiological functions [114]. Any related therapies would have to be restricted to the pancreas to avoid off-target effects.

### 5.3 Maintenance of cellular ATP levels

It has been shown previously and there is currently a consensus on the role of ATP loss in AP as result of intracellular Ca^2+^ overload causing mitochondrial dysfunction and cellular necrosis [3,92]. Therefore, providing the source of intracellular ATP, *i.e.*, pyruvate [59] could reduce pathology.

Our recent data [60] have shown that all tested agents, known to induce AP, significantly inhibit glucose metabolism by blocking hexokinases, leading to reduced production of pyruvate, fuel for mitochondrial ATP production and, therefore, together with inhibited glycolysis, result in intracellular ATP depletion. Combination of two pathological mechanisms:  Ca^2+^ overload and ATP depletion result in a positive amplification loop. While cytoplasmic Ca^2+^ is elevated, intracellular Ca^2+^ stores become empty due to substantial ATP depletion. Sustained elevation of cytoplasmic Ca^2+^and/or empty Ca^2+^ stores result in the instability of secretory granules [55], vacuole formation and subsequent intracellular activation of trypsinogen. Fortunately, the vicious circle of Ca^2+^ overload and ATP depletion can be broken, not only by inhibitors of Ca^2+^ channels but also by providing additional ATP to the cell [56].

We have demonstrated [60] that the addition of energy supplements, particularly, the natural carbohydrate galactose can considerably reduce cell injury in AP, both *in vitro* and *in vivo* (Fig. 5). While inhibitors of Ca^2+^ release and Ca^2+^ entry channels have clear merit for their development as a treatment for AP, galactose, as a freely available product and part of some food products such as lacto-free dairy, has no known negative side effects in humans [115], [116], [117]. Galactose has been used previously in humans both orally (1.5g/kg per day over 18 weeks [118]) and intravenously as a routine liver function test (0.6g/kg [119]) without any reported adverse effects.

Therefore, galactose would be a potentially valuable therapy against AP, both orally and intravenously for severe cases. Galactose can also be used preventatively, in cases with a significantly increased risk of AP, *i.e.*, when using asparaginase-based drugs for ALL [120]. Galactose can also be used in combination with other prospective AP treatments, *i.e.*, Ca^2+^ channel inhibitors when they became available in the clinic.

## 6 Conclusions

Aberrant Ca^2+^ signalling in all four cell types of exocrine pancreas discussed in this review has a substantial impact on the development of AP. Initially, the disease originates in PACs as a result of intracellular Ca^2+^ overload, ATP loss and necrotic cell death triggered mainly by alcohol metabolites and bile acids. Both pathological stimuli also adversely disturb the normal function of PDECs by evoking sustained cytosolic Ca^2+^ elevations, reduction of ATP and bicarbonate secretion, resulting in the reduction of the luminal pH and transport, weakening of tight junctions and cell death. Due to leakage of kallikrein from necrotic acinar cells, the tissue and plasma levels of BK are significantly elevated, which is responsible for Ca^2+^ signalling and activation of PSCs, releasing NO and contributing to pancreatic pathology. ATP and ADP, released from necrotic acinar cells, activate purinergic receptors located on the plasma membrane of pancreatic macrophages, mediating the development of the inflammatory response in the pancreas.

Recently, there have been a substantial progress in developing therapeutic treatments for AP. While mechanistic details of the initiation of AP are still under investigation, the main pathological processes are undoubtfully Ca^2+^ overload and ATP depletion**.** Therefore, one of most attractive strategies to reduce Ca^2+^ overload is to inhibit Ca^2+^ entry using Orai1/CRAC channel inhibitors. However, reduction of Ca^2+^ release from the stores via modulation of IP_3_Rs and RyRs can also become a useful alternative approach**.** ATP depletion can be effectively reduced by supplementation with the natural carbohydrate galactose, that can help on its own or in combination with inhibitors of calcium overload.

## CRediT authorship contribution statement

**Julia V. Gerasimenko:** Conceptualization, Writing – original draft, Writing – review & editing. **Oleg V. Gerasimenko:** Conceptualization, Writing – original draft, Writing – review & editing.

## Declaration of Competing Interest

The authors of the manuscript entitled “The role of Ca^2+^ signalling in the pathology of exocrine pancreas” declare no conflict of interest.

## Data Availability

No data was used for the research described in the article.
